# Comparison of the Efficiency of *Lepidium sativum*, *Ficus carica*, and *Punica granatum* Methanolic Extracts in Relieving Hyperglycemia and Hyperlipidemia of Streptozotocin-Induced Diabetic Rats

**DOI:** 10.1155/2021/6018835

**Published:** 2021-12-21

**Authors:** Shimaa Ramadan, Amany Mohamed Hegab, Yahya S. Al-Awthan, Mohammed Ali Al-Duais, Ahmed A. Tayel, Mahmoud A. Al-Saman

**Affiliations:** ^1^Department of Industrial Biotechnology, Genetic Engineering and Biotechnology Research Institute, University of Sadat City, Sadat City, Egypt; ^2^Developmental Pharmacology Department, The National Organization for Drug Control and Research (NODCAR), Egypt; ^3^Department of Biology, Faculty of Science, University of Tabuk, 71491 Tabuk, Saudi Arabia; ^4^Biochemistry Department, Faculty of Science, University of Tabuk, 71491 Tabuk, Saudi Arabia; ^5^Faculty of Aquatic and Fisheries Sciences, Kafrelsheikh University, Egypt

## Abstract

**Background:**

Diabetes mellitus (DM) is a metabolic disorder characterized by high blood glucose levels that occurs either due to insufficient insulin production or mounting resistance to its action. The purpose of this study was to investigate if methanolic extracts of *Lepidium sativum* seeds, *Ficus carica*, and *Punica granatum* leaves had any effect on blood sugar levels in normal and streptozotocin (STZ) diabetic rats, as well as to explore the most effective extract.

**Method:**

Healthy male albino rats weighing 185-266 g were divided into nine groups of eight rats each: normal control, diabetic control, diabetic rats with dietary supplements of *L. sativum*, *F. carica*, and *P. granatum* methanolic extracts, and diabetics treated with insulin. All of the rats were fed on ordinary diet with nutritional pellets and were given water *ad libitum*. To induce diabetes, all animals were administered with STZ intraperitoneal injection at a dose of 60 mg/kg body weight. For five weeks, the crude plant extracts were given orally to various groups of rats at doses of one hundred and two hundred mg/kg body weight. After that, animal groups were sacrificed and blood samples were taken.

**Results:**

Phytochemical analysis revealed that the highest amounts of polyphenolic compounds were present in *L. sativum* seeds and *P. granatum* leaves, while leaves of *F. carica* showed the highest amounts of alkaloid and flavonoid content compared to other extracts. Oral administration of *F. carica* and *L. sativum* extracts at the dosage of 100 and 200 mg/kg significantly reduced glucose, lipid profile, kidney, and liver enzyme levels. A significant increase in HbAlc levels was also observed with *L. sativum* extract at a dose of 200 mg/kg compared to diabetic controls. Mellitus rats supplemented with 100 and 200 mg/kg methanolic extracts of *P. granatum* had higher serum triglycerides and lower serum low-density lipoprotein cholesterol (LDL-C) than normal control rats. *F. carica* extract is more effective than *L. sativum* and *P. granatum* extracts in the prevention and control of type 2 diabetes mellitus (T2DM) and its consequences.

## 1. Introduction

DM is a group of metabolic diseases that lead to high levels of glucose (hyperglycemia), which is caused either by insufficient insulin production or a lack of insulin response [1]. Most cases of DM fall into the three broad categories of type 1, type 2, and gestational diabetes. Type 1 diabetes results from the pancreas's failure to produce enough insulin due to the loss of beta cells [2]. T2DM results from insulin resistance, a condition in which cells fail to respond properly to insulin; the most common cause is a combination of excessive body weight and insufficient exercise [3]. Gestational diabetes is the third form, and it occurs when pregnant mothers with no previous history of diabetes develop high blood sugar levels [2]. Ischemic heart disease, stroke, erectile dysfunction, blindness, delayed wound healing, and micro- or macrovascular complications such as retinopathy, neuropathy, and nephropathy all conspire with diabetes in the long run [4]. Approximately 422 million people around the world have diabetes; most of them live in low- and middle-income countries and 1.6 million deaths are directly attributed to diabetes annually, as reported by the World Health Organization (WHO) from global statistics collected in 2020 (https://www.who.int/health-topics/diabetes). There is currently no effective treatment for DM available in modern medicine. Insulin and oral antidiabetic medication are used to treat diabetes. Oral hypoglycemic agents may cause many side effects; hence, there is a need to look for most recent search antidiabetic agents that have high clinical effects with adverse effects.

At present, it has been found that the antidiabetic drugs used in the long-term treatment are associated with varieties of toxicities that have a negative impact on development. Traditional medicinal plants with antidiabetic qualities can be a valuable beneficial tool for producing safer and more effective oral hypoglycemic medications; likewise, the discovery of antidiabetic drugs has turned to natural plant sources with few side effects [5]. The hypoglycemic activity of a number of medicinal plants and its products has been assessed and revealed in animal trials as well as in humans. The active chemical constituents isolated from these plants and which are responsible for hypoglycemic properties contain flavonoids, phenolic, and alkaloid compounds [6]. There are 45,000 plant species that have records of widespread use in the treatment of this symptom in the world.

*Lepidium sativum* is a fast-growing edible herbaceous plant from the Brassicaceae family [7]. The seeds are used in folk remedies. The seeds have thermogenic, aphrodisiac, ophthalmic, diuretic, abortive, and contraceptive properties in nature [8]. Phytochemicals such as phenols, terpenoids, alkaloids, flavonoids, and sulphur compounds are abundant in *L. sativum* seeds. Sativum seed oil is high in linolenic acid and has an ideal ratio of *ω*-3 and *ω*-6 fatty acids, both of that have radioprotective and chemopreventive properties [9]. The aqueous extract of *L. sativum* has been shown to have hypoglycemic properties in diabetics.

The fig tree, *Ficus carica* Linn. (Moraceae), is grown in tropical and subtropical countries around the world for its nutritional and therapeutic benefits [10]. The leaves, bark, buds, fruits, seeds, and latex of *F. carica* have traditionally been used to treat jaundice, diarrhea, nutritional anemia, skin disorders, stomachache, dysentery, ulcers, diabetes, kidney, and the liver illnesses, as well as have anticancer and anti-inflammatory properties [11]. The antidiabetic property of *F. carica* has been reported in alloxan-induced diabetic rats [12]. The antihyperlipidemic influence of *F. carica* has been reported in high-fat diet animal models [13].

Pomegranate, *Punica granatum* L. (Lythraceae), is a fruit that originally came in the region extending from Iran to northern India and has been fostered in the Mediterranean region since ancient times. Fruits are generally frequently eaten fresh or in the form of products such as juice and jam [14]. Punicalagin, anthocyanin, phenolic acids, nonphenolic acids, and tannins are abundant in fresh pomegranate juice [15]. Pomegranate's antidiabetic properties are attributed to its phenolic chemicals that have the potential to operate as extremely effective agents in decreasing diabetes risk factors. Pomegranate extracts have been studied for their ability to prevent disorder.

The focus of this research was to see if methanolic extracts of mature seeds of *L. sativum*, as well as leaves of *F. carica* and *P. granatum*, had a hypoglycemic impact on normal and STZ-induced diabetic rats and also to investigate the most effective extract.

## 2. Materials and Methods

### 2.1. Chemicals

Sigma Chemical Co. provided the STZ (St. Louis, MO, USA). The bulk of the chemicals and reagents were of analytical quality.

### 2.2. Plant Collection and Identification

In May 2018, mature *Lepidium sativum* seeds, *Ficus carica*, and *Punica granatum* leaves have been obtained from a marketplace in Cairo, Egypt. The seeds and leaves would be rinsed and air-dried at room temperature (RT). Grinding was used to pulverize the dried materials to a size of 60 meshes. The ground seeds and leaves were kept at 4°C in brown glass containers until the extraction technique could be carried out in the lab.

### 2.3. Sample Preparation and Extraction

Twenty grams of powdered seeds and leaves was extracted by shaking at 150 rpm for 24 h at RT with 100 mL of 80% methanol. In a Büchner funnel, the extracts were filtered using Whatman no. 1 filter paper. The residue was reextracted with 50 mL methanol and filtered, and the filtrates were collected and evaporated at 40°C in a flash evaporator (Büchi, Flavil, Switzerland). It was redried in desiccators under vacuum until it reached a consistent weight. The weights of the final extracts were recorded, and the residue was resuspended in the smallest amount of Tween 80 possible to achieve a consistent concentration throughout all extracts. The extracts were stored at 4°C until they were used.

### 2.4. Quantitative Analysis of Bioactive Compounds

#### 2.4.1. Alkaloid Content

The total alkaloids were quantified using the method specified by the extract (mg/mL), which was dissolved in concentrated HCl (2 N) and then filtered. One milliliter of this solution was transferred to a separatory funnel and washed by chloroform (10 mL). The pH of the solution was brought to a neutral level. The sample has been mixed with 5 mL of bromocresol green (BCG) solution and 5 mL of phosphate buffer (pH 4.7). The component was vigorously agitated, and the resulting complex was extracted using chloroform. The complex's absorbance was measured at 470 nm against a blank.

#### 2.4.2. Polyphenol Content

The Folin-Ciocalteu method was used to determine the amount of total phenolics in extracts; 200 mL of extract has been combined with 1.5 mL of 10% Folin-Ciocalteu reagent and left for 5 min, after which 1.5 mL of sodium carbonate solution (6%) was added. After 1.5 h at RT, the spectrophotometric absorbance at 765 nm was measured. Gallic acid was used as comparison regard. Samples were tested in triplicate and expressed as mg GAE/g dry weight [16].

#### 2.4.3. Flavonoid Content

The level of total flavonoids was measured using the aluminum chloride colorimetric technique developed by [17]. Each extract sample (mg/mL) was blended with 0.5 mL of distilled water (DW). After adding 0.075 mL of 5% sodium nitrite solution to the mixture and incubation for 6 min, 0.15 mL of 10% AlCl_3_ was assigned, shaken, and left standing for 6 min at RT prior to adding 0.5 mL of 1 M NaOH and diluting with 0.275 mL DW, shaking, and leaving to stand for 15 min. A spectrophotometer was used to test the absorbance of the reaction mixture against a blank at 500 nm. Quercetin was the gold standard. The flavonoid content was measured in milligrams of quercetin equivalent (QE) for each/g of dry weight.

### 2.5. Feeding Study

#### 2.5.1. The Animal Model

Seventy-two mature male albino rats assessed (185-266 g) were obtained from the animal house of the National Organization Control and Drug Research (NODCAR), Egypt. They were kept in wire cages individually in an air-conditioned location with a temperature of 22 ± 2°C, relative humidity of 60%, and a 12 : 12 h light*-*dark cycle. Each animal was raised on a daily diet *ad libitum* during the acclimatization period. All the experimental process was carried out in compliance with the Ethics Committee of the National Organization for Drug Control and Research, Egypt. The animals were divided into nine cluster groups of eight rats, two control groups (control normal group, STZ group) and seven treatment groups.

#### 2.5.2. Induction of DM

In adult rats, a new T2DM animal model was created by combining STZ and nicotinamide (NAD) administration. The rats given NAD (230 mg/kg, i.p.) 15 min before STZ (60 mg/kg, i.p.) developed moderately and stable diabetes. A singular intraperitoneal (i.p.) dose of a newly buffered (0.1 mol/L sodium citrate, pH 4.5) solution of STZ at a dose of 60 mg/kg body weight was used to induce DM. Blood was drawn from the rats' tail arteries 72 h after they were given STZ. After 2 weeks, rats with hyperglycemia (blood sugar levels higher than 250 mg/dL) have been chosen for the experiment [18].

#### 2.5.3. Experimental Design

Seventy-two rats were divided into nine groups (*n* = 8) as follows.

*Group I*: normal control (negative control, fed a regular diet).

*Group II*: positive control (diabetic untreated rats were fed a regular diet).

*Group III*: diabetic rats were fed a regular diet, plus the *F. carica* leaf extract at 100 mg/kg BW, daily for 5 weeks.

*Group IV*: diabetic rats were fed a regular diet, plus the *F. carica* leaf extract at 200 mg/kg BW, daily for 5 weeks.

*Group V*: diabetic rats were fed a regular diet, plus the *P. granatum* leaf extract at 100 mg/kg BW, daily for 5 weeks.

*Group VI*: diabetic rats were fed a regular diet, plus the *P. granatum* leaf extract at 200 mg/kg BW, daily for 5 weeks.

*Group VII*: diabetic rats were fed a regular diet, plus the *L. sativum* seed extract at 100 mg/kg BW, daily for 5 weeks.

*Group VIII*: diabetic rats were fed a regular diet, plus the *L. sativum* seed extract at 200 mg/kg BW, daily for 5 weeks.

*Group IX*: diabetic rats were fed a regular diet with standard insulin a daily dose of 1 unit/kg BW (subcutaneous), for 5 weeks.

These doses were chosen for this study based on the results of previous toxicological studies [19–21].

A regular diet was made according to [22], and the freshly prepared extracts were taken orally every day for five weeks. For an overnight starved animal, body mass and blood sugar levels were monitored once a week. The animals fasted for an overnight period at the end of the trials, and blood sample was collected for various biochemical examinations. At the end of the investigation, the rats fasted overnight. A heparinized glass capillary was used to obtain blood samples from the venous retroorbital under anesthesia [23]. Efforts were taken to minimize animal pain during the experimentation. Organs such as the liver and pancreas were dissected and quickly cleaned with ice-cold saline before being kept for biochemical analysis [24].

### 2.6. Biochemical Analysis

#### 2.6.1. Lipid Profiling and Liver and Kidney Functions in the Serum Samples

Spectrophotometric methods were used with commercially available kits (Asan Pharmaceutical, Seoul, South Korea) to quantify total cholesterol (TC), HDL cholesterol, and serum triglycerides (TG) [25]. According to Warnick et al. [26], LDL-cholesterol was calculated as follows: LDL‐cholesterol = TC − HDL − (TG/5).

The Spinreact kit (Spain) was used to assay aspartate aminotransferase (AST) and alanine aminotransferase (ALT) using colorimetric enzymatic methods developed by Reitman and Frankel [27]. The World Federation of Clinical Chemistry advised using a kinetic technique to measure serum alkaline phosphatase (ALP) (IFCC). The concentrations of urea and creatinine were determined using a colorimetric enzymatic approach based on Kaplan [28], using a Diamond Diagnostics kit (Egypt).

### 2.7. Nonenzymatic Antioxidant Assay

#### 2.7.1. Determination of Hepatic Tissue Reduced Glutathione (GSH) Activity

The GSH concentration in the liver tissue was measured using the method described by Ellman [29], with some modifications as indicated by Nurrochmad et al. [30].

The homogenate was centrifuged after being added 10% TCA Ellman's reagents (19.8 mg of 5,5-dithiobisnitro benzoic acid (DTNB) in 100 mL of 0.1% sodium citrate) and 3.0 mL of phosphate buffer were added to 1 mL of supernatant (0.2 M, pH 8.0). The generated color's absorbance was measured immediately at 412 nm. It was measured in milligrams of glutathione per g of tissue.

#### 2.7.2. Estimation of Hepatic Malondialdehyde (MDA)

Thiobarbituric acid reactive substance was assessed using a colorimetric test to determine the MDA amount in the liver homogenates, as previously described [31]. One mL of 20% trichloroacetic (TCA) was added to 0.5 mL of the liver homogenate to precipitate the protein, followed by 3 mL of 1% orthophosphoric acid (1% H_3_PO_4_) and 1 mL of 0.6% thiobarbituric acid (0.6 TBA) in a boiling water bath. The samples were extracted with n-butanol and centrifuged after cooling. The samples' absorbance was measured at 520 and 535 nm. 1,1,3,3-Tetraethoxypropane was employed as a standard. MDA concentrations were measured in nmol/g of tissue.

#### 2.7.3. Estimation of the Liver Nitrite and Total Nitrite/Nitrate Contents

Total nitrate/nitrite accumulation in the liver was measured using an indicator of nitric oxide (NO) production [32]. Prior to NO estimation, the liver homogenate was deproteinized by adding absolute ethanol to the double volume of the sample. Experiments were carried out by adding equal volumes of sample, saturated VCl_3_ solution (200 mg VCl_3_ (Sigma-Aldrich) in 25 mL of 1 M HCl), Griess reagents (1 : 1 mixture of N-(1-naphthyl) ethylenediamine in deionized H_2_O), and 2% sulfanilamide (Sigma-Aldrich) in HCl, and a colorimeter was used to determine the absorbance at 540 nm.

### 2.8. Enzymatic Antioxidant Assay

#### 2.8.1. Estimation of the Liver Catalase (CAT) Activity

Tissue CAT activity was determined using the method described by Scaglione et al. [33]. In living cells, the assay of CAT is based on its reaction with a known amount of H_2_O_2_ CAT enzyme, which breaks down hydrogen peroxide into O_2_ and H_2_O. This enzyme is primarily responsible for regulating the metabolism of hydrogen peroxide. The CAT is a widely distributed enzyme found in nearly all living organisms. Because it can decompose into multiple molecules of hydrogen peroxide, it has one of the highest turnover rates of any enzyme.

#### 2.8.2. Estimation of Protein Content in the Liver

Total protein concentration was estimated by the method of Lowry et al. [34]. To make the Biuret reagent, mix 0.5 mL of 1% CuSO_4_ with 0.5 mL of 2% sodium potassium tartrate, and then, add 50 mL of 2% NaCO_3_ and 0.1 N NaOH. After that, the mix was incubated for 10 to 15 min at RT before adding 20 *μ*L of 1.0 N Folin-Ciocalteu reagent. Pipetting was done after each addition to ensure that the samples were thoroughly mixed. Before measuring absorbance at 650 nm and blanking on the water only control, the color was allowed to develop for 30 min at RT.

### 2.9. Histopathological Examination of the Pancreas

For adequate fixation, pancreatic specimens were cut into slices and maintained in 10% formalin. These tissues were treated and paraffin waxed. Hematoxylin and eosin were used to stain sections ranging in thickness from 2 to 8 m. Eosin stains the cytoplasm pink, while hematoxylin dyes the nuclei blue. According to the method of examination, all stained slices of the pancreatic tissues were inspected under a microscope for circulatory disturbances, inflammation, degenerations, apoptosis, necrosis, and any other pathological alterations [35].

### 2.10. Statistical Evaluation

All of the data was analyzed using the SPSS software version 20. To find any significant differences, a one-way analysis of variance (ANOVA) was utilized. Each item was evaluated three times, and the primary values and standard deviation (SD) were calculated. The significance of the variable mean differences was determined using Duncan's multiple range tests (*p* ≤ 0.05) [36].

## 3. Results

### 3.1. Chemical Constituents of the Extracts

Table 1 displays the phytochemical analysis of three distinct methanolic extracts of *L. sativum* seeds, *F. carica*, and *P. granatum* leaves in quantitative terms.

When comparing the polyphenolic content in the different extracts, the standard gallic acid curve revealed that *P. granatum* had the highest concentration (14.024 mg GAE/g), followed by *L. sativum* with 13.752 mg GAE/g, and finally *F. carica* with 10.177 mg GAE/g. The methanolic extract of *F. carica* leaves had the highest flavonoid content (8.220 mg QE/g), followed by *L. sativum* (1.516 mg QE/g), according to the standard quercetin curve. On the other hand, the highest alkaloid concentrations were found in the methanolic extract of *F. carica* leaves (0.344 mg/g), followed by *P. granatum* (0.233 mg/100 g).

### 3.2. Rat Body Mass and Glucose Level

Table 2 shows the effect of orally administered methanolic extracts of *L. sativum* seeds and *F. carica* and *P. granatum* leaves at various doses on the weight gain changes in STZ-induced hyperlipidemia and normal healthy rats for five weeks of treatment. The rats fed on the normal diet (negative control group) had significantly better body mass after five weeks than most diabetic rat groups fed on the same diet. Diabetic rats administered methanolic extract of the *L. sativum* seeds orally at a daily dose of 200 mg/kg or standard insulin at a single daily dose of one unit/kg had significant increase in body mass after five weeks compared to the positive control group (*p* < 0.05). The body masses of the diabetic rats that were orally administered methanolic extracts of *P. granatum* at doses of 100 and 200 mg/kg and *F. carica* at a dose of 100 mg/kg also were significantly different (*p* < 0.05). A dose of *L. sativum* 200 mg/kg and standard insulin had similar effects on the body mass of rats; and oral administration of *L. sativum* methanolic extract was found to increase body overweight.

Table 3 shows the effect of orally administered methanolic extracts of *L. sativum* seeds and *F. carica* and *P. granatum* leaves at various doses on the blood glucose level in STZ-induced diabetic and normal healthy rats for five weeks of treatment.

As compared to the normal control group, the diabetic control group had a significant (*p* < 0.05) increase in blood glucose level starting from the first week and had a lower body weight. When rats were given one unit/kg of insulin, their blood sugar levels decreased significantly (*p* < 0.05) within the first week, while their body weight increased significantly (*p* < 0.05) when compared to the positive control group. Insulin's significant effect on both variables persisted until the end of the experiment.

Methanol extracts of *F. carica* and *L. sativum* at a dose of 100 and 200 mg/kg, respectively, were administered to the groups. If compared to the diabetic control group, a significant reduction in blood glucose levels was observed from the first week to the end of the experiment, despite an increase in body weight alterations.

### 3.3. Serum TC, LDL, and HDL Levels

Serum cholesterol levels in diabetic rats showed a slight elevation, in comparison to the values detected in nondiabetic control rats (105.20 ± 7.29 vs. 99.20 ± 7.73 mg/dL) (Table 4). Cholesterol level increased in insulin-diabetic rats (119.33 ± 23.54 mg/dL) compared with the normal control rats. Slight reduction in cholesterol levels was observed after administration of all methanol extracts in diabetic rats except for *F. carica* at a dose of 200 mg/kg (107.00 ± 8.51 mg/dL).

Serum triglycerides were also significantly elevated in diabetic rats when compared to nondiabetic animals (197.60 ± 82.32 and 86.60 ± 5.94 mg/dL, respectively). Diabetic rats orally administered with methanol extract of *F. carica* at doses of 100 and 200 mg/kg showed significant decrease (*p* < 0.05) of plasma triglycerides. Diabetic rats supplemented with orally administered methanol extracts of *P. granatum* at doses of 100 and 200 mg/kg had higher levels of plasma triglycerides and lower plasma low-density lipoprotein cholesterol (LDL-C) than normal control. Serum high-density lipoprotein cholesterol (HDL-C) concentration did not alter with the administration of all methanol extracts to diabetic rats in comparison to positive control and nondiabetic control rats (*p* > 0.05).

### 3.4. Evaluation of the Liver and Kidney Functions and Glycosylated Hemoglobin (HbA1c)

Table 5 shows that diabetic rats had higher levels of AST and ALT liver enzymes (250.32 and 120.4 U/L, respectively) compared to normal rats and *L. sativum* 100 mg/kg (138.44 and 158.20 and 18.60 and 54.52 U/L, respectively). The liver enzymes, on the other hand, decreased in group IX rats who were only given insulin. Toxic renal consequences are often evidenced by a rise in serum urea and creatinine levels. For all methanol extract experiments with rats, the reported values of renal parameters (urea and creatinine) show a reduction. As a result, the diabetic group had a higher level of urea value than the control group. In comparison to the normal and diabetic groups, the insulin group had a higher level of creatinine.

The current study's findings show a decrease in HbA1c levels in the *F. carica* and *P. granatum* groups. The treatment of diabetic rats with *L. sativum* seed extract significantly (*p* < 0.05) increased the effect of STZ on HbAlc levels, as demonstrated by groups V and VI. Furthermore, diabetic rats treated with *F. carica* at a dose of 100 mg/kg improved their HbA1c levels more than the control group. Insulin, the standard treatment, had no effect on HbA1c levels.

ALP levels were significantly higher in the diabetic control group compared to the normal control group, while it was significantly lower in the experimental groups using *F. carica* at 100 mg/kg and *L. sativum* at 200 mg/kg compared to the diabetic control group. There were also no significant differences between the experimental groups.

### 3.5. Evaluation of Enzymatic and Nonenzymatic Antioxidant Parameters

The effect of the different methanol extracts of *L. sativum* seeds and *F. carica* and *P. granatum* leaves on the antioxidant marker levels of NO and MDA and the activity of antioxidant enzymes CAT and GSH is shown in Table 6.

When the diabetic positive control group was compared to normal control group rats, there was a significant decrease in the CAT activity (*p* > 0.05) and NO level. In the diabetic control group, even so, despite this, no significant (*p* > 0.05) increase in MDA and GSH content was observed. Once compared to animals in the normal and diabetic control groups, there was a significant decrease in NO levels in animals treated with insulin (*p* < 0.05) and all extracts of *F. carica* and *P. granatum* at a dose of 200 mg/kg. Except for *F. carica* at a dose of 100 mg/kg, no significant (*p* > 0.05) increase in MDA level was observed in the animals receiving the various treatments. Similarly, rats given 200 mg/kg doses of *F. carica* and *P. granatum* extracts exhibited a significant increase in CAT activity. GSH content was significantly increased (*p* < 0.05) by methanol extracts of *L. sativum*, particularly at doses of 100 mg/kg.

### 3.6. Pancreatic Histopathology Examination

**Figure 1:**
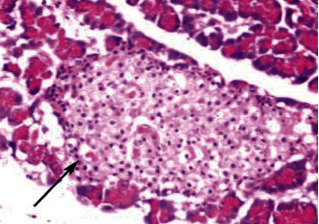
Photomicrograph of pancreatic tissue of control animal showing intact pancreatic islet (arrow) (H&E) (×400).

**Figure 2:**
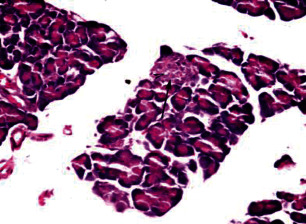
Photomicrograph of pancreatic tissue of STZ animal showing pancreatic islet reduced in size and cellularity with vacuolation between cells (arrowhead) (H&E) (×400).

**Figure 3:**
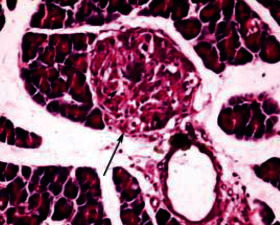
Photomicrograph of pancreatic tissue of insulin-treated animal showing pancreatic islet mildly restores its size and cellularity (arrow) with vacuolation in between cells (arrowhead) and congested blood capillary (double arrowhead) (H&E) (×400).

**Figure 4:**
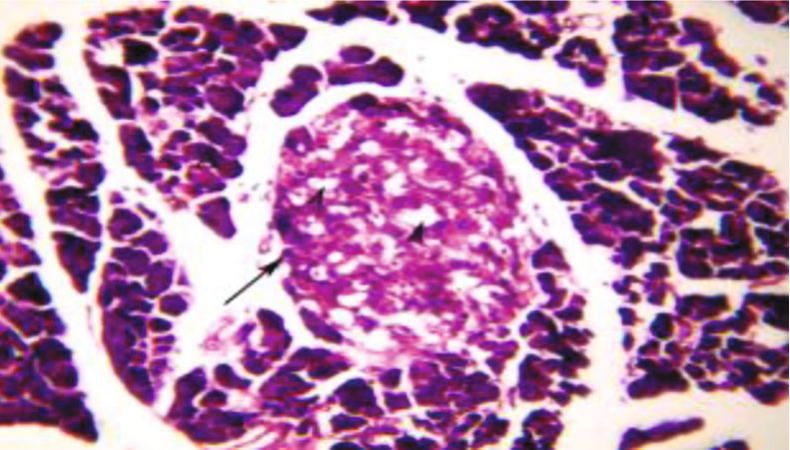
Photomicrograph of pancreatic tissue of *P. granatum* (100 mg/kg BW)-treated animal showing pancreatic islet (arrow) with vacuolation in between cells (arrowhead) and atrophied pancreatic acini (A) (H&E) (×400).

**Figure 5:**
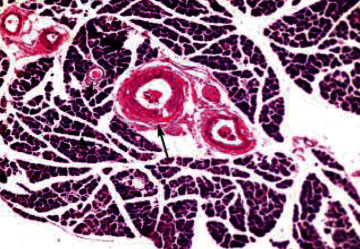
Photomicrograph of pancreatic tissue of *P. granatum* (200 mg/kg BW)-treated animal showing atrophied pancreatic acini (A), pancreatic duct (arrowhead), and blood vessel with thick wall (arrow) (H&E) (×100).

**Figure 6:**
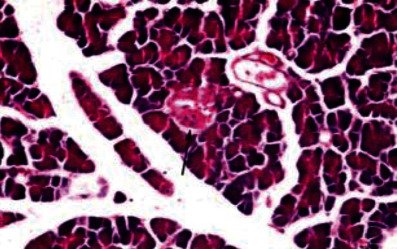
Photomicrograph of pancreatic tissue of *F. carica* (100 mg/kg BW)-treated animal showing pancreatic islet reduced in size and cellularity with vacuolation in between cells (arrowhead) (H&E) (×400).

**Figure 7:**
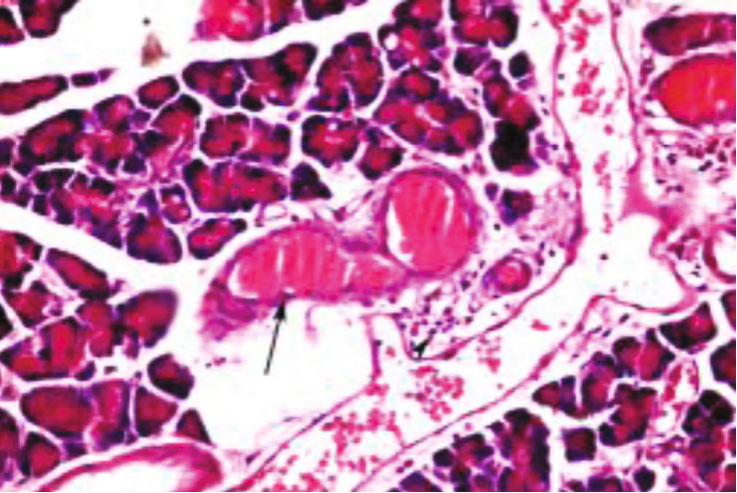
Photomicrograph of pancreatic tissue of *F. carica* (200 mg/kg BW)-treated animal showing inspissated pancreatic duct (arrow) and perivascular inflammatory infiltration (arrowhead) (H&E) (×400).

**Figure 8:**
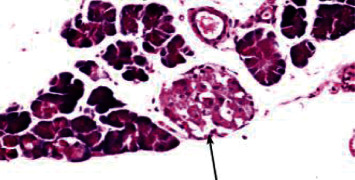
Photomicrograph of pancreatic tissue of *L. sativum* (100 mg/kg BW)-treated animal showing pancreatic islet (arrow) with severe vacuolation in between cells and hypocellularity (H&E) (×400).

Histological analysis of pancreatic islets indicated intact, undamaged pancreatic islets in the nondiabetic group (Figure 1). Additionally, typical pancreatic acini with vesicular nuclei and a normal pancreatic duct were observed in pancreatic tissue. Langerhans islets were found to be in good condition, containing differentiated cells. Microscopical analysis of pancreatic tissues (Figure 2) from the STZ group revealed a significant reduction in the size and quantity of islets of Langerhans, as well as modest degenerative alterations in the acini and minor perivascular inflammatory cells. Meanwhile, the pancreatic tissue insulin-treated group showed a slight improvement in pancreatic islet numbers and cellularity after five weeks (Figure 3). Atrophied pancreatic acini, pancreatic duct, and blood vessel with thick wall were observed in the *P. granatum* (100 mg/kg BW and 200 mg/kg BW) groups (Figures 4 and 5). The pancreatic islet size and cellularity (Figures 6 and 7) were reduced in *F. carica* (100 mg/kg BW) STZ-treated animals, with vacuolation between cells. Despite this, the *F. carica* group revealed a reduced islet of Langerhans size and quantity, as well as mild to moderate vacuolation in and between its cells, as well as inflammatory in filtrate cells surrounding blood vessels after a dose administration (200 mg/kg BW). Diabetic rats (Figure 8) treated with a methanolic extract of *L. sativum* (100 mg/kg BW) showed severe edema. Meanwhile, diabetic STZ rats that were given *L. sativum* (200 mg/kg BW) showed significant improvements in pancreatic islet tissues and healthy pancreatic ducts.

## 4. Discussion

The purpose of this research was to assess the hypoglycemic activity of methanol extracts of *F. carica*, *P. granatum*, and *L. sativum*. STZ-induced hyperglycemia was investigated using a reproducible experimental animal model. In this study, a significant decrease in blood glucose was observed in diabetic rats cured with *F. carica* and *L. sativum* at doses of 100 and 200 mg/kg, respectively, for 5 weeks. Previously, medicinal plants such as *F. carica*, *L. sativum*, and *P. granatum* have been discovered to have potential chemical constituents for hyperglycemia and antihyperlipidemia *via* increased insulin sensitivity and antioxidant mechanisms. Alkaloids, saponins, and flavonoids were found in high concentrations in plant ethanol extracts as functional molecules, Alkaloids, which are heterocyclic nitrogenous compounds, may play a role in diabetic disorders by inhibiting hyperglycemia and endogenous insulin excretion *via* inhibition of intestinal R-glucosidase [37].

Reduced blood sugar levels may enhance body weight in STZ-diabetic rats [38]. STZ raises blood glucose levels while decreasing insulin and C-peptide levels in rats.

This could be due to the extract's capacity to increase pancreatic secretion of insulin from regenerated cells or its ability to release bound insulin from regenerated cells by inhibiting ATP sensitive K^+^ channels. This one had a high concentration of phenolic compounds. Previous research found that phenolic composites acted on ATP-sensitive K^+^ channels and regulated blood glucose levels [39].

Hypercholesterolemia was a principal cause of atherosclerosis and coronary heart failure disease, both of which were secondary diabetes difficulties [40]. Two hundred mg/kg *L. sativum* and 100 mg/kg *F. carica* significantly reduced total cholesterol and LDL in STZ-diabetic rats. As a result, it is reasonable to conclude that *F. carica* and *L. sativum* may modulate lipid abnormalities in the blood [41].

The current study found an increase in lipid peroxidation and a decrease in GSH, a nonenzymatic antioxidant enzyme that participates in free radical scavenging in diabetes. Tissue injury is thought to be caused by the peroxidation of unsaturated fatty acids attacking membranes [42, 43]. Finally, lipid peroxidation results in significant membrane injury and dysfunction [44]. Pharmacological treatment could help prevent diabetes complications by lowering lipid peroxidation and improving antioxidant status [45].

According to our findings, *L. sativum* and *F. carica* significantly reduced the amplified lipid peroxidation, which can be attributed to the antioxidant effect of flavonoids, alkaloids, and total phenol detected in the preliminary phytochemical screening of the extracts. An increased body mass of diabetic-treated rats could be attributed to better glycemic control and increased structural protein biosynthesis [46].

In patients with the liver disease, the serum hepatic enzymes AST, ALT, and ALP are useful biomarkers of hepatic enzymes. The livers of diabetic rats treated with STZ have necrotized. The increased activities of AST and ALT in plasma could be attributed to the leakage of these enzymes from the liver cytosol into the bloodstream, indicating that STZ has a hepatotoxic effect [45, 47].

Treatment of diabetic rats with *F. carica*, *L. sativum*, and *P. granatum* at the dosages used moderated the activity of those enzymes in plasma compared to the diabetic untreated group and thus alleviating the liver damage caused by STZ-induced diabetes [48].

Renal parameter urea and creatinine levels in hyperglycemic rats were altered after administration of hetero alcoholic extract of *F. carica* in various dosages [43].

*L. sativum*[49] and *P. granatum* [50, 51] significantly decreased blood glucose levels when compared to controls, with enhanced body weight, which could be attributed to better control of the diabetic rats' hyperglycemic state [48].

High food consumption and declining body weight were observed in STZ-induced diabetic rats, indicating polyphagia and the weight loss due to extreme breakdown of tissue proteins [45, 52], as well as reduced function due to glucotoxicity [53].

Diabetic rats may gain less weight when treated with STZ due to dehydration and catabolism of fats and protein reactions, and muscle wasting may also explain why diabetic rats gain less weight [54]. Rats were in contrast to control animals in the experiments. They showed a significant decrease in MDA and NO. CAT levels in diabetic rats were found to be significantly lower after treatment with *F. carica*, *L. sativum*, and *P. granatum* compared with the control group. The analysis report of *L. sativum*, *F. carica*, and *P. granatum* showed the presence of phenol, alkaloid, and flavonoid content. Thus, the significant antidiabetic effect of *L. sativum* and *F. carica* may be due to the presence of polyphenol, alkaloid, and flavonoid constituents which alone or in synergism can impart therapeutic effect.

Plants' role in the prevention and treatment of mellitus is not limited to medicinal applications. The substantial body of literature has emerged linking the rising prevalence of diabetes to a decrease in the amount of vegetative matter absorbed in the diet [55].

The hypoglycemic action of *L. sativum* and *F. carica* had positive effects on blood glucose levels in this study, which is consistent with previous findings [56]. Diabetic rats were treated for two weeks with a methanol fraction of *F. mucoso* at the prescribed dose. Similarly, Pwaniyibo et al. [41] claim that the oral glucose tolerance of methanol *F. perifolia* leaves improved after 28 days of STZ in the animal models. As regards [45], it was revealed that cress seeds (CS) or *L. sativum* seeds alleviate hyperglycemia and oxidative stress damage in hyperglycemic rats.

Arafa et al. [57] found that the crude extract of *F. carica* is helpful in obesity-related diabetes and has outstanding therapeutic potential in high-fat diet-STZ-induced diabetic rats by controlling glucose, lipid metabolic, and oxidative stress variables.

The potential action of *L. sativum* and *F. carica* was comparable to that of the insulin reference medication used to treat diabetes. This could explain why *L. sativum* and *F. carica* have strong antioxidant and antidiabetic properties. The overall findings of this experimental study indicated that *L. sativum* and *F. carica* had strong antioxidant and antidiabetic properties. The findings of the study could lead to the expansion of pharmaceutical treatments for T2DM.

## 5. Conclusion

Diabetes treatment with synthetic drugs is expensive in developing countries due to poverty and a lack of access to medical care. As a result, phytotherapy has a significant role to play in developing countries when compared to synthetic drugs because it is safe, less expensive, and readily available as a natural gift. Data from our study confirm that the methanolic extracts of *F. carica* leaves and *L. sativum* seeds at a dose of 200 mg/kg have been found effective in regulating body mass, blood glucose, plasma creatinine, and plasma ALP levels, thereby showing improved renal function and hepatoprotective activity, also maintaining the lipid profile in the normal range and reducing the increased levels of plasma NO and MDA. The presence of phenolic compounds and flavonoids in *L. sativum* and *F. carica* extracts may be responsible for their hypoglycemic properties.

Also, it could be concluded that diabetic rats supplemented with orally administered methanol extracts of *P. granatum* had higher plasma triglycerides levels but lower plasma LDL-C levels than normal control. In addition, a significant increase in HbAlc levels was observed with *L. sativum* extracts compared to negative and positive controls. More clinical research is needed to establish the extracts' antidiabetic capabilities.

## Figures and Tables

**Table 1:** Polyphenol, flavonoid, and alkaloid content of *Lepidium sativum*, *Punica granatum*, and *Ficus carica* methanolic extracts.

	Phytoconstituents		
Plant	Polyphenol content (mg GAE/g DW)	Flavonoid content (mg QE/g DW)	Alkaloid content (mg/g DW)
*L. sativum*	13.752 ± 0.96^a^	1.516 ± 0.82^b^	0.138 ± 0.56^b^
*P. granatum*	14.024 ± 0.64^a^	1.286 ± 0.38^b^	0.233 ± 0.34^ab^
*F. carica*	10.177 ± 0.42^b^	8.220 ± 0.53^a^	0.344 ± 0.45^a^

**Table 2:** Effect of different *Lepidium sativum*, *Ficus carica*, and *Punica granatum* methanolic extracts on 5-week body mass, in STZ-induced diabetic rats.

#	Group type	Number of weeks and body weight of rats (g)					
		*W*_0_	*W*_1_	*W*_2_	*W*_3_	*W*_4_	Final
I	Control	226.4 ± 37.35	249.4 ± 45.48	257.4 ± 46.70	265.2 ± 46.68	270.6 ± 50.82	272.2 ± 58.37
II	STZ	206.6 ± 30.89	221.2 ± 36.89	229.8 ± 41.40	219.2 ± 34.79	220.4 ± 36.43	210.2 ± 38.81^∗^
III	*F. carica* 100 mg/kg	215.0 ± 30.43	232.4 ± 26.26	239.6 ± 30.39	247.6 ± 41.26	250.4 ± 44.21	257.8 ± 41.91
IV	*F. carica* 200 mg/kg	213.0 ± 44.08	207.0 ± 44.87	217.4 ± 41.46	212.8 ± 44.26	213.6 ± 44.18	212.6 ± 49.07^∗^
V	*L. sativum* 100 mg/kg	228.2 ± 08.61	220.0 ± 31.72	220.6 ± 27.53	227.0 ± 33.23	229.0 ± 38.78	231.8 ± 38.75
VI	*L. sativum* 200 mg/kg	221.2 ± 20.14	229.8 ± 15.88	251.6 ± 19.17	258.4 ± 21.22	269.0 ± 24.81	274.4 ± 25.66
VII	*P. granatum* 100 mg/kg	238.6 ± 18.58	234.4 ± 29.59	219.6 ± 38.39	245.4 ± 34.44	237.4 ± 35.96	233.6 ± 41.72
VIII	*P. granatum* 200 mg/kg	232.8 ± 24.09	239.0 ± 31.98	251.6 ± 43.42	256.8 ± 38.70	254.4 ± 40.56	249.8 ± 47.66
IX	Insulin	195.0 ± 28.52	211.8 ± 18.39	241.6 ± 20.24	245.6 ± 17.07	247.0 ± 23.97	274.0 ± 05.66

Values are given as mean ± SD significantly different at *p* < 0.05 for groups of nine animals each. ^∗^Statistically significant compared to the corresponding value in group I. ^**#**^Statistically significant compared to the corresponding value in group II.

**Table 3:** Effect of different *Lepidium sativum*, *Ficus carica*, and *Punica granatum* methanolic extracts on 5-week blood glucose, in STZ-induced diabetic rats.

#	Group type	Blood sugar mg/dL					
		*W*_0_	*W*_1_	*W*_2_	*W*_3_	*W*_4_	Final
I	Control	73.80 ± 7.05	81.4 ± 11.82	78.4 ± 9.32	71.4 ± 4.83	77 ± 2.12	76.8 ± 5.97
II	STZ	195.74 ± 118.81	311.2 ± 96.63	434.2 ± 158.35^∗^	352.4 ± 126.07^∗^	343 ± 115.79^∗^	284.4 ± 151.82
III	*F. carica* 100 mg/kg	228.56 ± 147.96	169 ± 142.28	187.4 ± 195.82	187.2 ± 214.15	200.92 ± 205.56	182.5 ± 175.92
IV	*F. carica* 200 mg/kg	245.04 ± 85.92	369.2 ± 200.12^∗^	233.1 ± 170.17	181.6 ± 140.31	300.6 ± 121.64^∗^	292.2 ± 133.74
V	*L. sativum* 100 mg/kg	291.20 ± 97.09^∗^	372.2 ± 171.37^∗^	174.6 ± 82.85	400.8 ± 118.87^∗^	410 ± 223.36^∗^	349.2 ± 160.4
VI	*L. sativum* 200 mg/kg	219.20 ± 61.26	172.4 ± 70.53	97 ± 29.49^**#**^	134.94 ± 37.21	155.6 ± 49.01^**#**^	161.8 ± 65.85
VII	*P. granatum* 100 mg/kg	285.40 ± 36.60^∗^	345.2 ± 111.82	256 ± 172.06	282.16 ± 148.32	253.6 ± 104.64	283.2 ± 147.11
VIII	*P. granatum* 200 mg/kg	271.80 ± 44.46^∗^	344.6 ± 110.91	341 ± 212.65	196 ± 131.33	230.89 ± 147.41	267.4 ± 236.29
IX	Insulin	255.40 ± 81.40^∗^	192.2 ± 156.73	256.2 ± 142.25	277.4 ± 116.5	254 ± 126.45	141.33 ± 22.19

Values are given as mean ± SD significantly different at *p* < 0.05 for groups of nine animals each. ^∗^Statistically significant compared to the corresponding value in group I. ^**#**^Statistically significant compared to the corresponding value in group II.

**Table 4:** Effect of different *Lepidium sativum*, *Ficus carica*, and *Punica granatum* methanolic extracts on serum cholesterol, triglycerides, cholesterol high-density lipoprotein (HDL), and cholesterol low-density lipoprotein (LDL) in STZ-induced diabetic rats after 5 weeks.

#	Group type	Lipid profile			
		Cholesterol (mg/dL)	Triglyceride (mg/dL)	HDL (mg/dL)	LDL (mg/dL)
I	Control	99.20 ± 7.73	86.60 ± 5.94	24.80 ± 2.05	60.00 ± 6.78
II	**STZ**	105.20 ± 7.29	197.60 ± 82.32^∗^	26.00 ± 2.74	50.40 ± 8.65
III	*F. carica* 100 mg/kg	94.40 ± 6.77	72.00 ± 10.93^**#**^	24.60 ± 2.07	56.20 ± 3.90
IV	*F. carica* 200 mg/kg	107.00 ± 8.51	75.20 ± 8.79^**#**^	27.80 ± 1.92	56.80 ± 7.95
V	*L. sativum* 100 mg/kg	98.00 ± 7.42	172.40 ± 70.40	26.00 ± 2.92	44.00 ± 6.40
VI	*L. sativum* 200 mg/kg	97.80 ± 8.41	175.80 ± 51.59	24.40 ± 2.79	41.20 ± 16.72
VII	*P. granatum* 100 mg/kg	99.00 ± 8.03	219.40 ± 48.00^∗^	26.00 ± 2.92	28.00 ± 4.30^∗^^**#**^
VIII	*P. granatum* 200 mg/kg	98.40 ± 5.41	215.20 ± 55.58^∗^	27.80 ± 2.49	37.40 ± 7.23^∗^
IX	Insulin	119.33 ± 23.54	112.33 ± 20.98	30.33 ± 7.57	66.67 ± 20.79

Values are given as mean ± SD significantly different at *p* < 0.05 for groups of nine animals each. ^∗^Statistically significant compared to the corresponding value in group I. ^**#**^Statistically significant compared to the corresponding value in group II.

**Table 5:** Effect of different *Lepidium sativum*, *Ficus carica*, and *Punica granatum* methanolic extracts on HbA1c, kidney functions (urea and creatinine), and the liver enzymes AST, ALT, and ALP, in STZ-induced diabetic rats after 5 weeks.

#	Group type	Parameters					
		HbA1c (%)	Urea (mg/dL)	Creatinine (mg/dL)	AST (U/L)	ALT (U/L)	ALP (U/L)
I	Control	11.75 ± 2.72	33.42 ± 05.68	0.58 ± 0.12	138.44 ± 043.16	18.60 ± 04.74	313.0 ± 88.35
II	STZ	13.20 ± 2.02	86.92 ± 24.12^∗^	0.70 ± 0.07	250.32 ± 102.98	120.4 ± 44.26	1160.4 ± 480.12^∗^
III	*F. carica* 100 mg/kg	09.60 ± 1.54	55.22 ± 10.72	0.65 ± 0.09	242.12 ± 221.30	98.34 ± 122.23	313.2 ± 104.63^**#**^
IV	*F. carica* 200 mg/kg	16.93 ± 1.96	76.40 ± 30.44	0.70 ± 0.09	250.40 ± 162.60	135.12 ± 126.23	781.4 ± 422.66
V	*L. sativum* 100 mg/kg	19.73 ± 3.3^#∗^	40.20 ± 07.22	0.62 ± 0.18	158.20 ± 14.31	54.52 ± 7.25	955.4 ± 593.90
VI	*L. sativum* 200 mg/kg	20.00 ± 4.14^#∗^	42.80 ± 07.01	0.58 ± 0.15	170.34 ± 21.24	52.18 ± 11.58	261.0 ± 37.97^**#**^
VII	*P. granatum* 100 mg/kg	14.26 ± 1.28	74.50 ± 35.50	0.66 ± 0.17	156.60 ± 27.17	61.00 ± 23.91	653.2 ± 186.48
VIII	*P. granatum* 200 mg/kg	16.64 ± 3.13	65.20 ± 36.74	0.78 ± 0.15	207.40 ± 78.12	72.40 ± 42.56	541.4 ± 488.61
IX	Insulin	20.96 ± 3.08^∗^#	73.20 ± 12.80	1.20 ± 0.26^#∗^	119.93 ± 23.07	35.93 ± 25.84	393.0 ± 78.24

Values are given as mean ± SD significantly different at *p* < 0.05 for groups of nine animals each. ^∗^Statistically significant compared to the corresponding value in group I. ^**#**^Statistically significant compared to the corresponding value in group II.

**Table 6:** Effect of different *Lepidium sativum*, *Ficus carica*, and *Punica granatum* methanolic extracts on the liver NO, MDA, CAT, and GSH in STZ-induced diabetic rats after 5 weeks.

#	Group type	Antioxidant parameters			
		NO (nmol/mL)	MDA (nmol/mL)	CAT (U/mL)	GSH (nmol/mL)
I	Control	95.23 ± 5.94	28.85 ± 4.6	123.96 ± 7.02	151.59 ± 3.42
II	STZ	54.29 ± 4.69^∗^	38.69 ± 7.06	60.33 ± 4.68^∗^	168.64 ± 6.33
III	*F. carica* 100 mg/kg	27.11 ± 5.01^∗^^**#**^	56.83 ± 4.68^∗^^**#**^	79.21 ± 25.47^∗^	149.91 ± 4.48
IV	*F. carica* 200 mg/kg	26.03 ± 5.22^∗^^**#**^	27.48 ± 4.34	103.91 ± 11.04^**#**^	155.68 ± 5.83
V	*L. sativum* 100 mg/kg	54.05 ± 5.96^∗^	18.76 ± 5.14^**#**^	73.96 ± 7.57^∗^	211.36 ± 22.92^∗^^**#**^
VI	*L. sativum* 200 mg/kg	63.11 ± 6.46^∗^	32.48 ± 2.35	78.37 ± 10.38^∗^	167.18 ± 8.94
VII	*P. granatum* 100 mg/kg	64.47 ± 3.33^∗^	28.50 ± 6.2	74.45 ± 26.19^∗^	168.09 ± 17.35
VIII	*P. granatum* 200 mg/kg	27.91 ± 6.70^∗^^**#**^	28.97 ± 6.77	115.32 ± 21.74^**#**^	157.73 ± 7.41
IX	Insulin	39.37 ± 5.54^∗^^**#**^	28.04 ± 6.66	84.44 ± 8.58^∗^	149.77 ± 3.35

Values are given as mean ± SD significantly different at *p* < 0.05 for groups of nine animals each. ^∗^Statistically significant compared to the corresponding value in group I. ^**#**^Statistically significant compared to the corresponding value in group II.

## Data Availability

All data are available in the manuscript and they are showed in figures and tables.
